# Analysis of Pork in Beef Sausages Using LC-Orbitrap HRMS Untargeted Metabolomics Combined with Chemometrics for Halal Authentication Study

**DOI:** 10.3390/molecules28165964

**Published:** 2023-08-09

**Authors:** Anjar Windarsih, Nor Kartini Abu Bakar, Dachriyanus, Nancy Dewi Yuliana, Florentinus Dika Octa Riswanto, Abdul Rohman

**Affiliations:** 1 Department of Chemistry, Faculty of Science, Universiti Malaya, Kuala Lumpur 50603, Malaysia; anjarwindarsih2@gmail.com (A.W.); kartini@um.edu.my (N.K.A.B.); 2 Research Center for Food Technology and Processing (PRTPP), National Research and Innovation Agency (BRIN), Yogyakarta 55861, Indonesia; 3 Faculty of Pharmacy, Andalas University, Padang 25175, Indonesia; dachriyanus@phar.unand.ac.id; 4 Department of Food Science and Technology, IPB University, Bogor 16680, Indonesia; nancy_dewi@apps.ipb.ac.id; 5 Halal Science Center, IPB University, Bogor 16129, Indonesia; 6 Division of Pharmaceutical Analysis and Medicinal Chemistry, Faculty of Pharmacy, Campus III Paingan, Universitas Sanata Dharma, Yogyakarta 55282, Indonesia; dikaocta@usd.ac.id; 7 Department of Pharmaceutical Chemistry, Faculty of Pharmacy, Universitas Gadjah Mada, Yogyakarta 55281, Indonesia; 8 Center of Excellence, Institute for Halal Industry and Systems (PUIPT-IHIS), Universitas Gadjah Mada, Yogyakarta 55281, Indonesia

**Keywords:** beef sausages, pork, LC–HRMS metabolomics, PLS-DA, halal authentication

## Abstract

Beef sausage (BS) is one of the most favored meat products due to its nutrition and good taste. However, for economic purposes, BS is often adulterated with pork by unethical players. Pork consumption is strictly prohibited for religions including Islam and Judaism. Therefore, advanced detection methods are highly required to warrant the halal authenticity of BS. This research aimed to develop a liquid chromatography–high-resolution mass spectrometry (LC–HRMS) method to determine the halal authenticity of BS using an untargeted metabolomics approach. LC–HRMS was capable of detecting various metabolites in BS and BS containing pork. The presence of pork in BS could be differentiated using principal component analysis (PCA) and partial least squares-discriminant analysis (PLS-DA) with high accuracy. PLS-DA perfectly classified authentic BS and BS containing pork in all concentration levels of pork with R^2^X = (0.821), R^2^Y(= 0.984), and Q^2^ = (0.795). The level of pork in BS was successfully predicted through partial least squares (PLS) and orthogonal PLS (OPLS) chemometrics. Both models gave high R^2^ (>0.99) actual and predicted values as well as few errors, indicating good accuracy and precision. Identification of discriminating metabolites’ potential as biomarker candidates through variable importance for projections (VIP) value revealed metabolites of 2-arachidonyl-sn-glycero-3-phosphoethanolamine, 3-hydroxyoctanoylcarnitine, 8Z,11Z,14Z-eicosatrienoic acid, D-(+)-galactose, oleamide, 3-hydroxyhexadecanoylcarnitine, arachidonic acid, and α-eleostearic acid as good indicators to detect pork. It can be concluded that LC–HRMS metabolomics combined with PCA, PLS-DA, PLS, and OPLS was successfully used to detect pork adulteration in beef sausages. The results imply that LC–HRMS untargeted metabolomics in combination with chemometrics is a promising alternative as an analytical technique to detect pork in sausage products. Further analysis of larger samples is required to warrant the reproducibility.

## 1. Introduction

The growth of the halal industry has increased over the past few decades around the world, not only in Muslim countries but also in non-Muslim countries [1]. Among the halal industry products, halal foods have become the most important thing since the demand for halal foods has significantly increased throughout the year [2]. This has been triggered by the changing human lifestyle because halal food is closely associated with a healthy lifestyle. For certain religions such as Islam and Judaism, consuming halal foods is a must. According to Islam’s sharia law, consuming foods containing non-halal components such as pork and its derivatives, alcohol, etc., is strictly forbidden. However, some cases of food products adulteration by intentionally adding non-halal substances have been reported [3,4]. Beef sausages (BS), one of the most favored meat products, are often subjected to adulteration with pork due to the difference between the price of beef and pork meats to gain more profits. Unfortunately, detection of pork in such processed foods is not an easy task. Visual observation and simple detection methods are almost unable to recognize and detect pork in processed foods [5]. As a consequence, halal authentication of food products is negligible to warrant its halal authenticity from adulteration of non-halal components.

Currently, various detection methods using different approaches are being developed to acquire effective and efficient analytical methods for analysis of non-halal components in food products. In most countries, methods based on protein detection using ELISA (enzyme-linked immunosorbent assay) and DNA detection using polymerase chain reaction (PCR) and quantitative PCR (qPCR) have been widely developed and used [6,7]. Other methods, such as gas chromatography and liquid chromatography combined with advanced detectors such as mass spectrometry [8,9], vibrational spectroscopy [10] including mid-infrared, near infrared, and Raman spectroscopy, as well as nuclear magnetic resonance spectrometry [11], have also been developed for halal detection and authentication. Among the methods used for halal authentication, the qPCR method has become the method of choice in countries, including in Indonesia, Malaysia, Thailand, and others, for analysis of non-halal substances in food products [6,12]. However, for certain cases such as highly processed food products, the non-halal DNA degrades and is unable to be amplified. There is also a big challenge in isolating DNA from highly processed foods where only very low levels of DNA are present [4,13]. In addition, this requires rigorous sample preparation steps and the high cost of a DNA isolation kit [14]. Therefore, it is important that other approaches be developed to obtain effective analytical techniques for analysis of non-halal components in highly processed meat products. With the development of omics-based science, the metabolomics approach offers versatility for food authentication purposes, including halal authentication of food products [15,16]. Previous study on pork authentication in sausages has been successfully carried out using densitometry analysis along with the analytical method of validation [17]. With the higher interest in chemometrics-assisted halal authentication, other studies have reported the implementation of Fourier-transform infrared spectrophotometry combined with chemometrics for analyzing lard in sausage samples [18].

Metabolomics is a study of the identification of metabolites in samples using certain approaches [19,20]. Untargeted metabolomics has been proven to have a capability to identify various metabolite compositions in certain samples [21]. It has been used in various field of research including food analysis and authentication because of its ability to determine metabolite composition in foods. The changes in metabolites’ composition in food products due to factors such as adulteration, contamination, and substitution can be observed using a metabolomics approach [22,23]. Several analytical techniques, such as gas chromatography and liquid chromatography coupled with mass spectrometry detection and nuclear magnetic resonance spectrometry (NMR), have been utilized for metabolomics research [24,25]. The liquid chromatography–high-resolution mass spectrometry (LC–HRMS) technique is a powerful analytical method for its untargeted metabolomics analysis of food products due to its high specificity and high reliability. Metabolites are separated through LC systems prior to being measured using an HRMS detector, therefore, larger number of metabolites can be acquired [26].

Due to the large dataset resulting from untargeted LC–HRMS metabolomics, an adequate statistical analysis such as chemometrics is required. Chemometrics consists of pattern recognition and multivariate calibration, which could include multivariate data from metabolomics analysis [27,28]. Principal component analysis (PCA) and partial least squares-discriminant analysis (PLS-DA) have been widely applied to sample differentiation in metabolomics analysis. In addition, partial least squares (PLS) and its orthogonal form (OPLS) of regression have also been used for food analysis [29]. Several studies on food authentication using a combination of LC–HRMS and chemometrics have been reported, such as evaluation of metabolite changes of duck breast meat subjected to various preservation times [30]. Metabolomics using LC–HRMS and chemometrics has also been used to identify metabolite changes of low-temperature sausages stored at room temperature [31]. It has also been utilized to investigate the changes of lipid metabolites in Hengshan goat sausages during preservative treatment [32]. In addition, LC–HRMS metabolomics and chemometrics have been used for detection of pork in raw beef meat [22] and tuna meat [33], and the presence of pork in beef meatballs has been successfully detected by LC–HRMS non-targeted metabolomics and chemometrics [34].

However, limited studies have been conducted on metabolomics analysis using LC–HRMS for halal authentication of meat products. There is no report on the study of halal authentication of sausages using LC–HRMS metabolomics and chemometrics. As a consequence, a wide opportunity is still open to explore and develop a metabolomics analysis system using LC–HRMS for halal authentication study of meat products. Therefore, the general objective of this study was to develop an untargeted LC–HRMS metabolomics analysis for halal authentication testing of BS adulterated with pork. Moreover, the specific purposes were to apply chemometrics analysis to both pattern recognition and multivariate calibration for detection of pork adulteration in BS. In this study, metabolomics was used to uncover various metabolites in beef and pork sausages using simple preparation techniques. In addition, the present study performed identification of discriminating metabolites’ potential as biomarkers to discriminate beef and pork sausages using chemometrics techniques, providing useful information for detecting pork adulteration in BS products.

## 2. Results and Discussion

### 2.1. Untargeted Metabolomics Analysis

**Figure 1:**
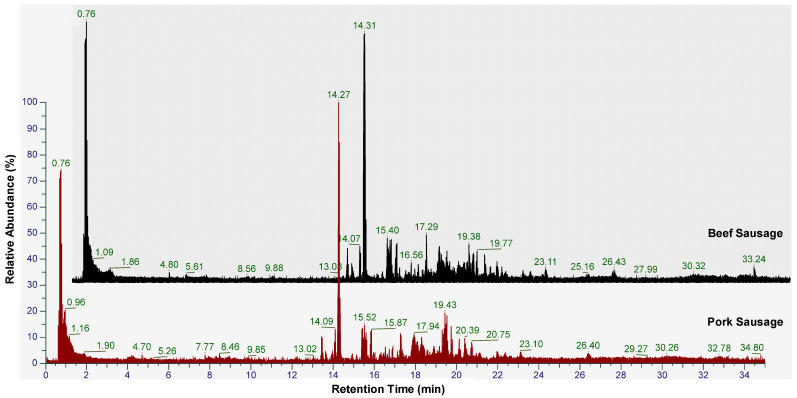
The total ion chromatogram obtained from LC–HRMS measurement of beef sausage and pork sausage.

A metabolomics approach using high-resolution mass spectrometry coupled with liquid chromatography could be used to separate and identify metabolites extracted from sausages made from either beef or pork. The untargeted workflow could be utilized for identification of the many metabolites in sausages. In this study, an enormous number of detected metabolites were found in both positive and negative ionization modes. Figure 1 illustrates the results of total ion chromatogram measurement of BS, PS, and BS containing pork. After filtering the metabolites, 302 metabolites were selected and used for further chemometrics analysis. The metabolites were dominated by amino acids and lipids, as it is known that meat contains a large proportion of amino acids and lipids. Metabolites-based discrimination for differentiating pork and beef sausages was previously performed by evaluating the fatty acid content. The pork sausages contained the fatty acids palmitic acid (37.75%), oleic acid (25.29%), myristic acid (22.24%), and lauric acid (8.46%). On the other hand, the fatty acid content of beef sausages was dominated by palmitic acid (42.31%), oleic acid (20.19%), stearic acid (10.92%), and myristic acid (7.66%). The metabolites contained in sausages made from beef, pork, and a mixture of beef and pork were varied. The difference of the peak area from each metabolite among samples indicated the changes of metabolites due to pork adulteration in BS. This information is very useful in differentiating BS, PS, and BS adulterated with pork for halal authentication purposes. Using ANOVA post hoc testing, 71 significant metabolites (*p* < 0.05 and fold change > 2) were identified. Identification of metabolites is very useful for differentiation of certain samples for authentication purposes. Previous studies have reported the use of an untargeted approach in metabolomics analysis using LC–HRMS for analysis of meat metabolites such as beef, pork, and chicken [35,36]. In addition, it has also been used for metabolomic profiling of meat products such as ground meats, meatballs, and sausages [31,37].

### 2.2. Chemometrics Analysis: Supervised and Unsupervised Pattern Recognition

**Figure 2:**
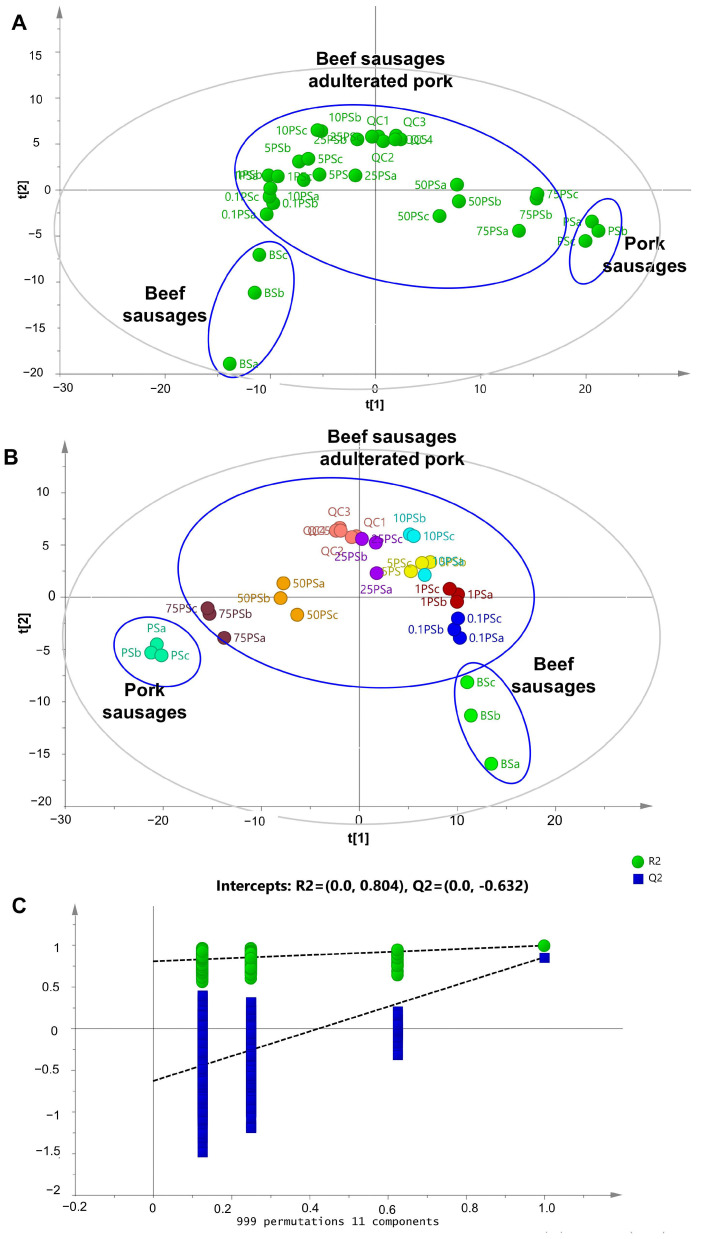
Differentiation of beef sausages, beef sausages containing pork, and pork sausages using principal component analysis (**A**), partial least squares-discriminant analysis (**B**), and permutation tests from PLS-DA (**C**) (BS = beef sausage; 0.1 PS = 0.1% pork; 1 PS = 1% pork; 5 PS = 5% pork; 10 PS = 10% pork; 25 PS = 25% pork; 50 PS = 50% pork; 75 PS = 75% pork; PS = pork sausage; QC = quality control).

The first pattern recognition applied to differentiate BS and BS containing pork was PCA. The score plot result of PCA using six principal components (PCs) indicated the differentiation results of BS, PS, and BS containing pork (Figure 2A). Unit variance scaling or autoscaling was applied to the metabolite dataset to obtain better variation of the dataset. The PCA could clearly separate pure BS and pure PS into different clusters. Meanwhile, the BS containing pork appeared between the cluster of pure BS and pure PS. Good model fitness was obtained in PCA, indicated by its R^2^ (0.710), whereas the Q^2^ value (0.40) indicated good predictive capacity. As an unsupervised pattern recognition analysis, PCA works by reducing the number of original variables used into several PCs in which the first two PCs contain the largest variation. PCA has been commonly used to identify patterns from metabolomics measurement, including in meat differentiation [38]. Trivedi et al. [22] reported the use of PC for differentiation of pure beef meat and meat adulterated with pork in raw meat samples. The metabolites used for PCA were obtained from GC-MS analysis. The utilization of PCA for the detection of adulteration in chevon, chicken, donkey, and beef meat has been reported by Akhtar et al. [11] using ^1^H-NMR metabolomics data. However, the number of metabolites obtained from ^1^H-NMR were quite a bit lower than those found using the LC–HRMS technique.

Besides PCA, the metabolomics data were subjected to PLS-DA analysis, a type of supervised pattern recognition technique to discriminate and classify samples. Using four latent variables, the sausage samples could be correctly discriminated and classified into their corresponding classes (Figure 2B). The samples of BS adulterated with pork were successfully classified into their concentration levels of pork. In addition, BS containing a low level of pork (0.1%, 1%, and 5%) could be clearly discriminated from pure BS, indicating high accuracy of the PLS-DA model to detect pork adulteration in BS. The accuracy of the PLS-DA model was also confirmed by its R^2^X (0.821) and R^2^Y (0.984) values. Meanwhile, the good predictive ability of the PLS-DA model was illustrated by its Q^2^ value (0.795). As the pork concentration increased in BS, the score plots were moving away from those of pure BS but closer to those of PS, indicating changes in the metabolomic composition in BS due to the addition of pork.

**Figure 3:**
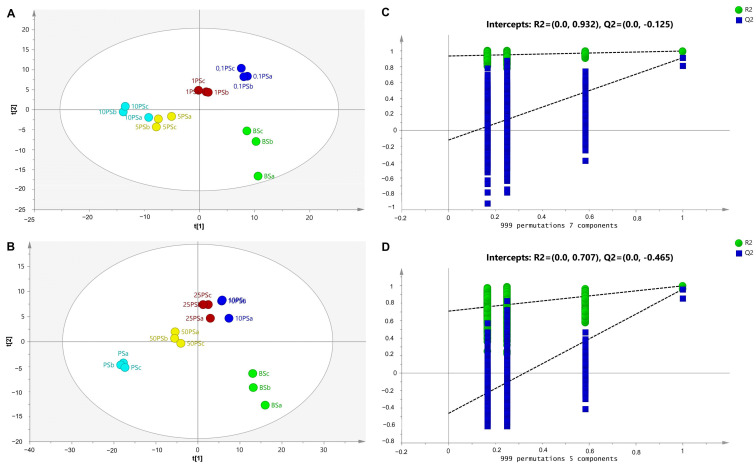
Partial least squares-discriminant analysis (PLS-DA) to discriminate pure beef sausage from beef sausage containing pork in low concentration (**A**), a high concentration of pork (**B**), permutation testing from the PLS-DA model using a low concentration of pork (**C**), and using a high concentration of pork (**D**) (BS = beef sausage; 0.1 PS = 0.1% pork; 1 PS = 1% pork; 5 PS = 5% pork; 10 PS = 10% pork; 25 PS = 25% pork; 50 PS = 50% pork; PS = pork sausage).

To obtain a clearer discrimination in sausage samples with a low concentration of pork, PLS-DA was used on pure BS and BS containing low amounts of pork (0.1–10%), performed applying seven LV (latent variables). Figure 3A depicts the score of PLS-DA that demonstrates a perfect discrimination among sample classes. The BS sample containing 0.1% pork could be clearly separated from the pure BS sample, indicating the good ability of PLS-DA to detect pork in BS presented in low concentration, associated with the high sensitivity of the developed model. The PLS-DA found R^2^X = 0.790, R^2^Y = 0.997, and Q^2^ = 0.943. In addition, PLS-DA for discrimination of BS with high levels of pork, excluding BS samples with a low concentration of pork, was also performed. As shown in Figure 3B, using five LV, BS containing 10%, 25%, and 50% pork could be very clearly separated from authentic BS. Good model accuracy and good predictive capacity were obtained, as indicated by the values of R^2^X (0.738), R^2^Y (0.984), and Q^2^ (0.847).

Such validation tests were applied to warrant the performance of the PLS-DA model, including cross-validation tests and permutation tests. In addition, a receiver operating characteristics (ROC) test was also a powerful indicator used to validate the PLS-DA model [39]. The leave-one-out cross-validation test confirmed the accuracy and precision of the developed model. A permutation test applied for 999 permutations and 11 components revealed the highest original Q2 value among all permutated Q2 values, indicating the validity of the PLS-DA model using all samples (Figure 2C). In addition, the Q2 intersection was between zero and lower than zero (0.0, −0.632), which was also an indicator of model validity through permutation testing. The permutation test applied in the PLS-DA model of BS containing a low concentration of pork as well as BS containing a high concentration of pork also revealed the validity of the models (Figure 3B,D) with intersection of Q2 of (0.0, −0.125) and (0.0, −0.465), respectively. Another validation technique, the ROC test, was used to evaluate the models’ validity through evaluation of the AUC value from each sample class. When the AUC value is closer to 1, the model is valid because the error occurring in classification was low. In this study, the ROC value of each sample class observed in all three PLS-DA models was 1, indicating no misclassification occurred, which indicates valid models.

Previous research reported the use of PLS-DA for meat differentiation both in raw meat and in meat products. The adulteration of beef meat with pork could be clearly discriminated using PLS-DA created from lipid metabolites data. The PLS-DA could detect 10%, 25%, and 50% pork adulteration in beef [22]. In our present study, the PLS-DA could detect up to 0.1% pork adulteration in BS samples. In addition, PLS-DA using non-volatiles metabolomics data obtained from LC–HRMS untargeted metabolomics could discriminate low-temperature sausage samples stored at room temperature on different days. Samples stored at day 2 were not significantly different from those tested on day 0, however, samples at day 4, 6, 8, 10, and 12 were clearly discriminated from those of day 0 [31].

### 2.3. Identification of Discriminating Metabolites

**Figure 4:**
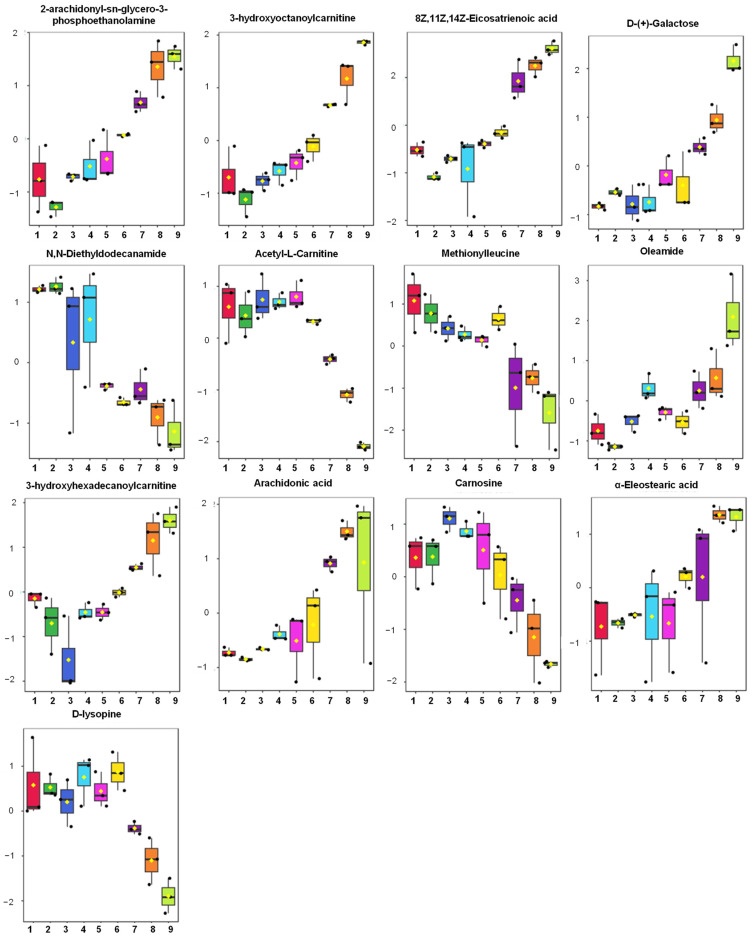
Box plot of discriminating metabolites with variable importance for projections (VIP) values larger than 2.0 to discriminate beef and pork sausages (the number in x axis shows sample classes of beef sausages: 1 = 0% pork, 2 = 0.1% pork, 3 = 1% pork, 4 = 5% pork, 5 = 10% pork, 6 = 25% pork, 7 = 50% pork, 8 = 75% pork, 9 = 100% pork; the y axis is the normalized peak area of metabolite).

**Figure 5:**
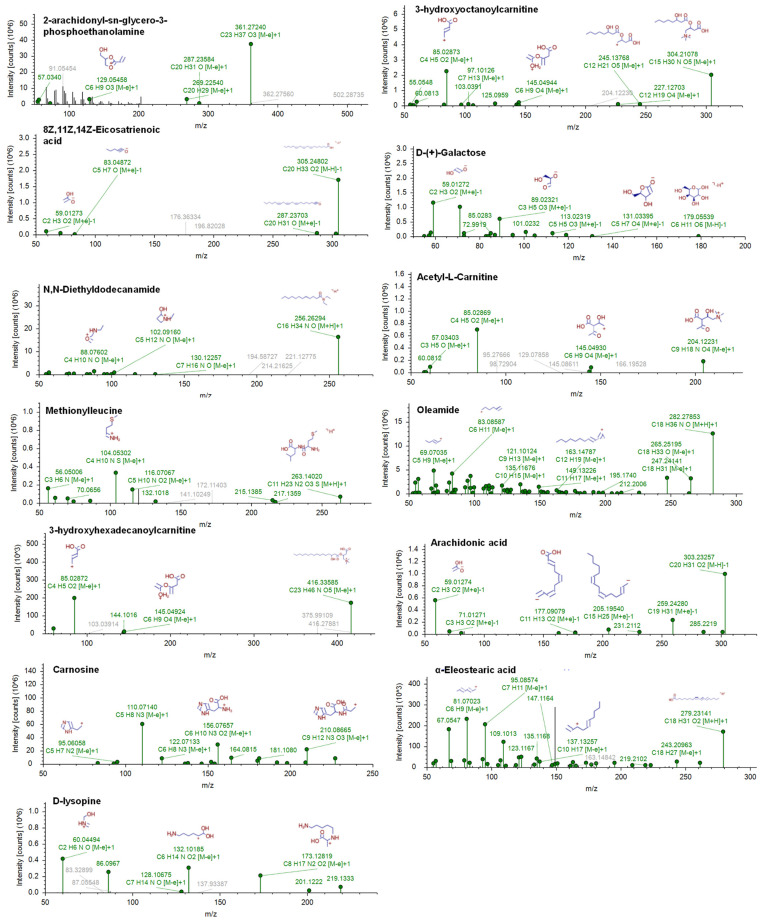
The mass spectra annotation and fragmentation pattern of discriminating metabolites having variable importance for projections (VIP) values larger than 2.0.

Variable importance for projections (VIP) analysis from PLS-DA was performed to identify the discriminating metabolites’ potential for biomarkers. The discriminating metabolites are believed to have important roles and contributions in sample discrimination [40]. Table S1 shows the discriminating metabolites with a VIP value of more than 1.5. The discriminating metabolites were varied, including amino acids, organic acids, peptides, fatty acids, and other lipids. These metabolites are very useful for further analysis in investigating biomarker candidates for discrimination of pork contained in BS samples. The higher the value of the metabolite’s VIP, the more relevant the variable for sample discrimination. Further investigation of the metabolites with a high VIP value (VIP ≥ 2.0) was performed, as depicted in the box plots (Figure 4). In addition, the annotation of metabolites with their mass fragmentation pattern is illustrated in Figure 5. The box plots revealed the changes of metabolites due to pork adulteration in each sample class. Metabolites of 2-arachidonyl-sn-glycero-3-phosphoethanolamine, 3-hydroxyoctanoylcarnitine, 8Z,11Z,14Z-Eicosatrienoic acid, D-(+)-Galactose, oleamide, 3-hydroxyhexadecanoylcarnitine, arachidonic acid, and α-eleostearic were found to be gradually increased in accordance with the increasing levels of pork contained in BS. Therefore, these metabolites can be used as indicators to discriminate pork from BS. Meanwhile, metabolites of N,N-diethyldodecanamide, acetyl-L-carnitine, methionylleucine, carnosine, and D-lysopine increased in high levels of beef meat. This stage is an important step to simplify the complex steps of biomarker investigation to identify potential candidates of biomarkers.

Metabolites of 8Z,11Z,14Z-eicosatrienoic acid, oleamide, arachidonic acid, and α-eleostearic are fatty acid compounds, the rich nutritional fatty acid contents contained in meats. Different types of meats contain different fatty acids which can be used to distinguish them [41]. Acylcarnitine metabolites such as 3-hydroxyoctanoylcarnitine, 3-hydroxyhexadecanoylcarnitine, and acetyl-L-carnitine are reported to be especially high in red meat. Acetyl-L-carnitine is high in red meat such as beef meat, and the content of acetyl-L-carnitine in beef meat is higher compared to chicken and pork meat [42]. Carnosine and methionylleucine are a dipeptide which can be found in meats. Carnosine naturally occurs in the skeletal muscle. It plays several roles, such as a neurotransmitter, a pH buffer in muscle, and an antioxidant to reduce the number of free radicals by direct reaction with reactive oxygen species (ROS). Meanwhile, methionylleucine is an incomplete breakdown products of protein catabolism. It has a role in cell-signaling effects [43]. D-lysopine is an amino acid derivative obtained from the L-lysine derivative. The muscle tissue of beef has a high content of D-lysopine, therefore in the present study, D-lysopine was found to be high in BS samples [44].

### 2.4. Chemometrics Analysis: Multivariate Calibration

**Figure 6:**
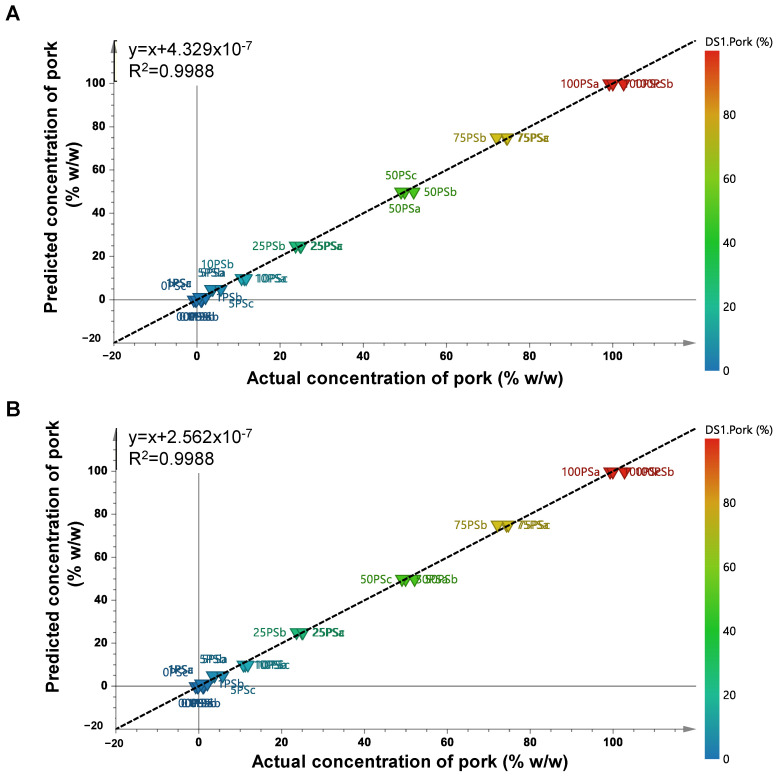
Partial least squares regression model (**A**) and orthogonal partial least squares regression model (**B**) for predicting pork in beef sausages (0 PS = 0% pork; 0.1 PS = 0.1% pork; 1 PS = 1% pork; 5 PS = 5% pork; 10 PS = 10% pork; 25 PS = 25% pork; 50 PS = 50% pork; 75 PS = 75% pork; 100 PS = 100% pork).

The metabolites obtained from VIP analysis (VIP > 1.0) were subjected to PLS and OPLS analysis to build a prediction model capable of detecting levels of pork in BS. Using three components, a high correlation between actual levels and predicted levels of pork was obtained in PLS. The obtained coefficient of determination (R^2^) was 0.9988, whereas the RMSEE (root mean square error of estimation) was 1.32% and RMSECV (root mean square error of cross-validation) was 3.16%. The OPLS also successfully predicted the levels of pork in BS with R^2^ = 0.9988, followed by its RMSEE = 1.32% and RMSECV 3.18%. Both prediction models of PLS and OPLS were comparable in terms of their performance. The PLS and OPLS plots of actual and predicted values of pork contained in BS are shown in Figure 6. The results of PLS and OPLS regression indicated that the metabolites with VIP values larger than 1.0 were good predictors for pork adulteration in BS with high accuracy and precision. It is known that PLS and OPLS techniques have become the most used multivariate regression because of their performance in providing high accuracy and precision in prediction models from multivariate data. These methods have wide applications in food authentication, such as predicting level of adulterants and contaminants in food products. Previous study has stated that the OPLS-VIP model provided a better diagnostic compared to PLS-VIP [45]. However, the ease of combining PLS and OPLS with chemical data obtained from various analytical techniques makes these methods the preferred choices for researchers [46].

## 3. Materials and Methods

### 3.1. Chemicals

The LC-MS grade acetonitrile, methanol, and water were supplied by Thermo Fisher (Thermo Scientific, Rockford, IL, USA). The hypergrade methanol for liquid chromatography and formic acid were purchased from Merck (Darmstadt, Germany). The ESI velos positive and ESI velos negative for calibration solution were obtained from Thermo Scientific (Rockford, IL, USA).

### 3.2. Meat Collection

The beef meat samples were collected from five different beef slaughtering houses in Yogyakarta and Central Java, Indonesia. Meanwhile, pork meat was purchased from three different pork meat sellers in Yogyakarta, Indonesia. The loin part was used in both types of meats. The meats were transported under frozen condition and immediately stored in a freezer at 20 °C prior to use for sausage preparation.

### 3.3. Sausage Formulation

The sausage samples were made using the ingredients of meat (beef and pork), tapioca flour, salt, onion, and pepper. The proportion of meat was 90% while the other portion (10%) consisted of tapioca flour, salt, pepper, and onion. Meat samples were sliced into small pieces and then ground using a meat grinder. Beef and pork meat were prepared in separate processes using a separate meat grinder. The ground meat was mixed with other non-meat ingredients and homogenized to obtain homogenous mixtures. The dough was then formed into sausages using a manual sausage maker. The adulterated BS sausage with pork was made by mixing beef sausage with pork using various concentration levels of pork (0.1%, 1%, 5%, 10%, 25%, 50%, and 75% *w*/*w*).

### 3.4. Extraction of Metabolites from Sausages

Amounts of 5 g of sausage sampled from pure BS, pure PS, and BS adulterated pork were analytically weighed and placed into a 50 mL falcon tube. Each sample was extracted with 25 mL of hypergrade methanol for liquid chromatography and vortex for 30 s. The mixture was then placed into a Beaker glass for homogenization using an UltraTurrax homogenizer (IKA, Guangzhou, China) operated at 5000 rpm for 5 min. The sample was then subjected for metabolite extraction using an ultrasonic (Elma Sonic, Singen, Germany) for 30 min at room temperature (25 °C). After the sonication process was completed, the sample was placed into a freezer (−20 °C) for 1 h to precipitate proteins. The sample was then taken and placed at room temperature (25 °C) for 20 min. Subsequently, the supernatant was taken by centrifugation at 5000 rpm at 4 °C; 1 mL of supernatant was pipetted into an HPLC vial for analysis using LC–HRMS. Amount of 100 µL was taken from each sample and collected into the same tube for preparing a quality control (QC) sample [30].

### 3.5. Metabolomics Analysis

Metabolomics analysis was performed according to the previous method by Windarsih et al. [30]. A sophisticated instrument of ultra high-performance liquid chromatography (UHPLC, Vanquish, Thermo Scientific, USA) connected to a high-resolution mass spectrometer (HRMS, Q-Exactive Orbitrap, Thermo Scientific, USA) was used for untargeted metabolomics analysis of sausages. A reverse-phase chromatographer using a C-18 column (Accucore, 10 cm × 2.1 mm × 2.6 µm) was applied to screen metabolite compositions in sausages. The mobile phase consisted of water (A) and methanol (B) containing 0.1% formic acid for each mobile phase. The analytes were subjected to a gradient elution technique as follows: 0–5 min (5% B), 5–20 min (5–90% B), 20–30 min (90% B), and 30–35 min (90% B–5% B). The sample was injected at 10 µL for each measurement both in blank and samples. During the analyte separation, the column temperature was maintained at 40 °C. Analytes were detected in an Orbitrap mass analyzer HRMS after eluting from the HPLC system. The HRMS parameters, such as sheath gas flow rate and auxiliary gas flow rate, were set at 32 arbitrary units (AU) and 8 AU, respectively. Electrospray ionization (ESI) was used in both positive and negative modes employing a spray voltage of 3.5 kV. Before the analysis, both ESI positive and ESI negative were calibrated using an ESI calibration solution. The temperature for capillary was set at 320 °C during the analysis. The compounds were scanned at mass ranges from 66.7 to 1000 m/z using resolution of 70,000 FWHM and 17,500 FWHM for MS1 and MS2, respectively. The collision energy applied was 10 NCE for both positive and negative ionization mode.

### 3.6. Data Processing

The raw data from LC–HRMS measurement producing a total ion chromatogram (TIC) was exported using XCalibur software (Thermo Scientific, USA). The TIC was then imported to Compound Discoverer software (Thermo Scientific, USA) for further processing such as spectrum selection, retention time alignment, background correction, feature detection, and compound identification. The identification of metabolites was performed via a search against the database from MzCloud (https://www.mzcloud.org/ accessed on 20 June 2023) and Chemspider (https://www.chemspider.com accessed on 20 June 2023). The selected metabolites were filtered according to the following criteria: metabolites having a full match with MzCloud and Chemspider, metabolites with data-dependent acquisition for preferred ions, and metabolites with a mass error between −5 ppm and 5 ppm. The results of the selected metabolites from Compound Discoverer were then exported to Microsoft Excel for chemometrics analysis.

### 3.7. Chemometrics Analysis

The data were subjected to sum normalization and continued for data scaling (auto scaling technique) prior to chemometrics analysis. The analysis was performed using SIMCA software 14.1 version (Umetrics, Umea, Sweden) and Metaboanalyst 5.0. Firstly, the data were subjected to principal component analysis (PCA), an unsupervised technique to identify natural samples grouping. The values of R^2^ and Q^2^ were recorded to evaluate the performance. Later, partial least squares-discriminant analysis (PLS-DA) was applied as a supervised technique for samples discrimination. The values of R^2^X, R^2^Y, and Q^2^ were recorded to observe the model performance. Such validation tests using the permutation test, ROC (receiver operating characteristics) test, and cross-validation were carried out to warrant the validity of the PLS-DA model to be free from overfitting. The identification of discriminating metabolites which have potential as biomarker candidates for sample differentiation was performed using variable importance for projections (VIP) value analysis. Metabolites with a VIP value higher than 1.0 were considered as discriminating metabolites.

Secondly, the data were subjected to multivariate calibration chemometrics using partial least squares (PLS) and orthogonal PLS (OPLS) to build prediction models for predicting the amount of pork added in beef sausages (BS). The PLS plots and OPLS plots of actual levels and predicted levels of pork were recorded. The performance of PLS and OPLS was observed using R^2^, RMSEE (root mean square error of estimation), and RMSECV (root mean square error of cross-validations).

## 4. Conclusions

Pork sausage and beef sausage containing pork could be classified and clearly discriminated from authentic beef sausages using partial least squares-discriminant analysis (PLS-DA) with high accuracy without misclassification. A number of discriminating metabolites with potential as biomarkers were obtained through VIP analysis. Metabolites of 2-arachidonyl-sn-glycero-3-phosphoethanolamine, 3-hydroxyoctanoylcarnitine, 8Z,11Z,14Z-eicosatrienoic acid, D-(+)-galactose, oleamide, 3-hydroxyhexadecanoylcarnitine, arachidonic acid, and α-eleostearic acid gradually increased along with the levels of pork added in BS, thereby providing good indicators to detect pork present in BS samples. In addition, partial least squares (PLS) and orthogonal analysis confirmed that metabolites with a VIP value > 1 were good indicators to predict pork in BS with high accuracy and precision. It can be concluded that an LC–HRMS untargeted metabolomics approach incorporating chemometrics analysis is very promising as a powerful tool for detecting pork and other non-halal meats in meat products by identifying their metabolomic composition. Further research on metabolomics using larger samples of beef and pork sausages is required to obtain more representative and reliable results. Verification of the discriminating metabolites is required, for example, by quantification using internal standards. Analysis using various commercial beef sausages targeting the discriminating metabolites is also important for further research.

## Data Availability

The data presented in this study are available on request from the corresponding author.

## Supplementary Materials

The following supporting information can be downloaded at: https://www.mdpi.com/article/10.3390/molecules28165964/s1, Table S1: Discriminating metabolites’ potential as biomarker candidates for discrimination of pork in beef sausage investigated using variable importance for projections of PLS-DA with a VIP more than 1.5.

## Sample Availability

Samples of the compounds are available from the authors.
