# Supplementary material for: Analysis of Pork in Beef Sausages Using LC-Orbitrap HRMS Untargeted Metabolomics Combined with Chemometrics for Halal Authentication Study
Source: Molecules. 2023 Aug 9;28(16):5964. doi: 10.3390/molecules28165964 (PMC10459517; doi:10.3390/molecules28165964)
Supplement: Supplementary file 1 [file molecules-28-05964-s001.zip › molecules-2497501-supplementary.pdf]

Supplementary material

Analysis of Pork in Beef Sausages using LC-Orbitrap HRMS Untargeted Metabolomics Combined with Chemometrics for Halal Authentication Study

Table S1. Discriminating metabolites potential for biomarker candidates for discrimination of pork in beef sausage investigated using variable importance for projections of PLS-DA with VIP more than 1.5

No.	Compounds	VIP	Formula	Calculated m/z	RT (min)
1	2-arachidonyl-sn-glycero-3-phosphoethanolamine	2.62	$C_{25}H_{44}NO_7P$	501.28405	18.063
2	3-hydroxyoctanoylcarnitine	2.61	$C_{15}H_{29}NO_5$	303.20397	7.982
3	8Z,11Z,14Z-Eicosatrienoic acid	2.49	$C_{20}H_{34}O_2$	306.25484	20.001
4	D-(+)-Galactose	2.43	$C_6H_{12}O_6$	180.06254	0.743
5	N,N-Diethyldodecanamide	2.40	$C_{16}H_{33}NO$	255.25549	13.786
6	Acetyl-L-carnitine	2.30	$C_9H_{17}NO_4$	203.11496	0.735
7	Methionylleucine	2.26	$C_{11}H_{22}N_2O_3S$	262.13459	5.189
8	Oleamide	2.19	$C_{18}H_{35}NO$	281.27061	19.323
9	3-hydroxyhexadecanoylcarnitine	2.18	$C_{23}H_{45}NO_5$	415.32865	16.184
10	Arachidonic acid	2.17	$C_{20}H_{32}O_2$	304.23968	19.454
11	Carnosine	2.12	$C_9H_{14}N_4O_3$	226.10579	0.682
12	α-Eleostearic acid	2.09	$C_{18}H_{30}O_2$	278.22392	17.165
13	D-lysopine	2.02	$C_9H_{18}N_2O_4$	218.12612	2.058
14	Hexanoylcarnitine	1.99	$C_{13}H_{25}NO_4$	259.17782	6.697
15	Monoolein	1.98	$C_{21}H_{40}O_4$	356.29111	19.599
16	(2R)-1-{[(2-Aminoethoxy)(hydroxy)phosphoryl]oxy}-3-hydroxy-2-propanyl (11Z)-11-icosenoate	1.97	$C_{25}H_{50}NO_7P$	507.33094	18.30
17	Palmitoleic acid	1.94	$C_{16}H_{30}O_2$	254.22403	19.179
18	Tiglylcarnitine	1.84	$C_{12}H_{21}NO_4$	243.14671	3.835
19	Decanoylcarnitine	1.82	$C_{17}H_{33}NO_4$	315.24023	12.838
20	Acetylcholine	1.82	$C_7H_{15}NO_2$	145.11001	0.722
21	DL-Carnitine	1.79	$C_7H_{15}NO_3$	161.10479	0.729
22	3-(2-methylpropyl)-octahydropyrrolo[1,2-a]pyrazine-1,4-dione	1.78	$C_{11}H_{18}N_2O_2$	210.13639	7.474
23	Undecanedioic acid	1.72	$C_{11}H_{20}O_4$	216.13548	12.667
24	3-Hydroxy-3-[(3-methylbutanoyl)oxy]-4-(trimethylammonio)butanoate	1.70	$C_{12}H_{23}NO_5$	261.15705	1.308
25	2-(acetylamino)-4-(methylthio)butanoic acid	1.69	$C_7H_{13}NO_3S$	191.06076	3.883
26	2-Acetyl-4-methylpyridine	1.69	C_8H_9NO	135.06833	1.059
27	(2R)-3-{[(2-Aminoethoxy)(hydroxy)phosphoryl]oxy}-2-hydroxypropyl (5Z,8Z,11Z)-5,8,11-Icosatrienoate	1.66	$C_{25}H_{46}NO_7P$	503.30000	18.657
28	C8-Carnitine	1.63	$C_{15}H_{29}NO_4$	287.20912	10.311

29	(15Z)-9,12,13-Trihydroxy-15-octadecenoic acid	1.61	$C_{18}H_{34}O_5$	330.24020	13.85
30	All-cis-4,7,10,13,16-Docosapentaenoic acid	1.55	$C_{22}H_{34}O_2$	330.25529	20.035
31	(1Z,2S)-N-[(2S,3R,4E,8E)-1,3-Dihydroxy-4,8-octadecadien-2-yl]-2-hydroxyundecanimidic acid	1.54	$C_{29}H_{55}NO_4$	481.41193	18.524
32	Myristyl sulfate	1.54	$C_{14}H_{30}O_4S$	294.18622	21.649
33	3-(Icosanoyloxy)-4-(trimethylammonio)butanoate	1.53	$C_{27}H_{53}NO_4$	455.39619	18.470
34	L-Histidine	1.52	$C_6H_9N_3O_2$	155.06919	0.777
35	Sphinganine	1.51	$C_{18}H_{39}NO_2$	301.29712	15.226
